# Interactive Effect of Salicylic Acid on Some Physiological Features and Antioxidant Enzymes Activity in Ginger (*Zingiber officinale* Roscoe)

**DOI:** 10.3390/molecules18055965

**Published:** 2013-05-21

**Authors:** Ali Ghasemzadeh, Hawa Z. E. Jaafar

**Affiliations:** Department of Crop Science, Faculty of Agriculture, University Putra Malaysia, 43400 Serdang, Selangor, Malaysia

**Keywords:** salicylic acid, nitrate reductase, peroxidase activity, superoxide dismutases, catalase enzyme, proline activity

## Abstract

The effect of foliar salicylic acid (SA) applications (10^−3^ and 10^−5^ M) on activities of nitrate reductase, guaiacol peroxidase (POD), superoxide dismutases (SOD), catalase (CAT) and proline enzymes and physiological parameters was evaluated in two ginger varieties (Halia Bentong and Halia Bara) under greenhouse conditions. In both varieties, tested treatments generally enhanced photosynthetic rate and total dry weight. Photosynthetic rate increases were generally accompanied by increased or unchanged stomatal conductance levels, although intercellular CO_2_ concentrations of treated plants were typically lower than in controls. Lower SA concentrations were generally more effective in enhancing photosynthetic rate and plant growth. Exogenous application of SA increased antioxidant enzyme activities and proline content; the greatest responses were obtained in plants sprayed with 10^–5^ M SA, with significant increases observed in CAT (20.1%), POD (45.2%), SOD (44.1%) and proline (43.1%) activities. Increased CAT activity in leaves is naturally expected to increase photosynthetic efficiency and thus net photosynthesis by maintaining a constant CO_2_ supply. Our results support the idea that low SA concentrations (10^–5^ M) may induce nitrite reductase synthesis by mobilizing intracellular NO^3−^ and can provide protection to nitrite reductase degradation *in vivo* in the absence of NO^3–^. Observed positive correlations among proline, SOD, CAT and POD activities in the studied varieties suggest that increased SOD activity was accompanied by increases in CAT and POD activities because of the high demands of H_2_O_2_ quenching.

## 1. Introduction

Salicylic acid (SA) is classified as a phenolic compound, a group of substances that can regulate plant growth [1]. SA application influences a wide variety of plant processes, including stomatal closure [2], plant growth and yield [3] and induction of antioxidant synthesis [4,5]. The known effects of SA on stomatal function, chlorophyll content, transpiration rate and respiratory pathways indicate that SA and related phenolic compounds may be involved in regulation of some photosynthetic reactions.

The effect of salicylic acid on plant physiological processes varies depending on species, developmental stage, SA concentration and environmental conditions [6]. Foliar application of SA (1.4 × 10^−4^ M) to *Brassica napus* was found to enhance chlorophyll concentration [7,8]. When SA was applied to leaves of *Phaseolus vulgaris* and *Commelina communis*, transpiration rates decreased [9,10]; in soybean and corn leaves [11], however, increases in stomatal conductance and transpiration rate were observed in response to spraying with SA, acetylsalicylic acid and gentisic acid. In addition, water use efficiency, rate of transpiration and internal CO_2_ increased in soybeans after supplementation with SA [12]. Similarly, barley seedlings exposed to SA for one week exhibited increases in CO_2_ compensation point and stomatal resistance, followed by a decrease of about 50% in ribulose-1,5-bisphosphate carboxylase/oxygenase (Rubisco) activity [13]. In the same study, an increase in phosphoenolpyruvate carboxylase levels and a decrease in photosynthetic rate were also noted. In contrast to the above observations, application of SA increased Rubisco activity and photosynthetic rate in maize and mustard plants [14]; in mustard plants, moreover, water use efficiency and carboxylation efficiency increased in connection with the high photosynthetic rate. 

Alonso-Ramirez *et al.* [15] reported that in *Arabidopsis* gibberellic acid stimulation may play an important role in SA biosynthesis and action, and that some of the physiological effects of this hormone may be mediated by SA. For instance, SA has been shown to have an important role in heat stress response [16] and in improved *Arabidopsis thaliana* seed germination under salt stress conditions [17]. Reactive oxygen species (ROS), including hydrogen peroxide (H_2_O_2_), superoxide (O^2−^), hydroxyl radical (HO^−^) and singlet oxygen, can disrupt normal plant metabolism through oxidative damage to proteins, lipids, nucleic acids, photosynthetic enzymes and pigments [18,19]. To overcome oxidative stress, plants have developed enzymatic and non-enzymatic antioxidant defense mechanisms to scavenge ROS [20]. The most important antioxidant enzymes are peroxidase (POD; EC 1.11.1.7), catalase (CAT; EC 1.11.1.6) and superoxide dismutases (SOD; EC 1.15.1.1). SODs convert O^2−^ into H_2_O_2_ and O_2_, with CAT and POD transforming H_2_O_2_ into H_2_O [21,22]. In addition, non-enzymatic antioxidative carotenoids, such as beta carotene and xanthophylls, can reduce ROS and stabilize photosynthetic complexes [23,24].

Ginger (*Zingiber officinale* Roscoe) is a traditional folk medicinal plant extensively used in Malaysian cooking. One of the most widely used herbs, especially in Asia, it contains several interesting bioactive constituents and possesses health-promoting properties. It is also an inexpensive and important food additive. In Malaysia, spices such as ginger are traditionally used in food preparations to improve flavor, but also play a major role in providing required antioxidants for the body. In previous studies, we uncovered potent antioxidant and anticancer activities in Malaysian young ginger (Halia Bara and Halia Bentong) [25,26,27]. Useful information regarding the effect of SA on growth of Malaysian ginger varieties, with their high levels of beneficial compounds, is still scarce, however.

The objective of this study was to examine the effect of SA foliar applications on net photosynthetic rate, stomatal conductance, intercellular carbon and biomass production in two varieties of Malaysian ginger, namely, Halia Bentong and Halia Bara. To determine the most effective SA concentration, we also investigated alterations in nitrate reductase, POD, SOD, CAT and proline activities. 

## 2. Results and Discussion

### 2.1. Effect of SA Concentration on Leaf Gas Exchange Parameters

**Figure 1:**
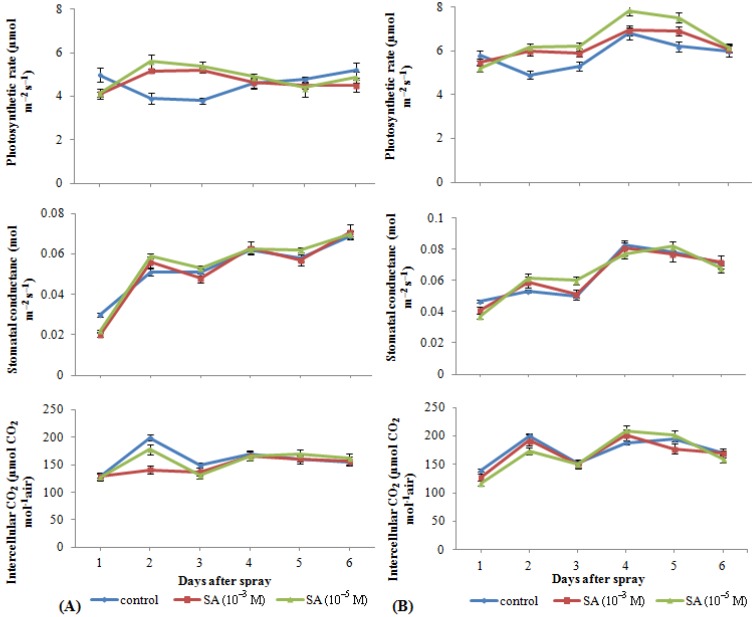
Effect of foliar application of salicylic acid on photosynthetic rate, stomatal conductance and intercellular CO_2_ (Ci) in ginger varieties ((**A**) Halia Bentong and (**B**) Halia Bara). Error bars indicate means of triplicate measurements ± SEM.

Two days after foliar application of 10^–5^ M SA, photosynthetic rate increases of 18% and 19.2% were observed in Halia Bara and Halia Bentong, respectively; spraying with 10^–3^ M SA resulted in 9.2% and 10.1% increases within the same time frame (Figure 1). On days 2 to 5 following either SA treatment, photosynthetic rate decreased significantly in Halia Bentong, followed by a slight increase on day 6, whereas photosynthetic rate in Halia Bara increased significantly from days 3 to 4 and then decreased. The highest photosynthetic rate in Halia Bara, 7.8 µmol m^–2^ s^–1^, was observed on day 4, while in Halia Bentong, the highest rate, 5.6 µmol m^–2^ s^–1^, was recorded 2 d after spraying. Stomatal conductance in treated plants generally increased or was the same as in control plants. Of the two SA treatments, plants of both varieties exposed to 10^−5^ M exhibited the highest photosynthetic rates. 

Induction of photosynthetic rate and stomatal conductance by SA has been previously demonstrated [3]. For example, Zhou *et al.* [28] found that stem injection of corn plants with 10^–2^ M SA increased photosynthetic rate. Stomatal regulation and behavior are very important factors controlling photosynthetic rate. Intercellular CO_2_ concentration (Ci) was lower in treated plants than in the controls (Figure 1). While increased photosynthetic rates seen 2 d after SA application were generally accompanied by increased or unchanged stomatal conductance levels and transpiration rates, intercellular CO_2_ concentrations of treated plants were generally lower than in control plants. If increased stomatal opening was the primary cause of increased photosynthetic activity, increases in internal carbon would be expected. These results thus suggest that increases in photosynthetic rate following SA spray applications were due to increased CO_2_ uptake at the chloroplast level rather than simple increases in stomatal opening, *i.e*., reduced resistance to entry of CO_2_ in the leaves. Increased photosynthetic rates accompanied by increases in stomatal conductance and lowered internal carbon values, as seen in our results, also imply that the photosynthetic rate increase may have been due to enzyme-related activities at the chloroplast level. Stomatal conductance readings, along with decreases in Ci values, strongly suggest that changes in photosynthetic rate were due to changes within the leaf rather than increased stomatal opening. 

In this study, we used a commercially feasible method of SA application—leaf spraying—to demonstrate how SA might be used to increase photosynthetic and growth rates in a wide range of crop plants. We observed that the lower SA concentration (10^–5^ M) was generally more effective in enhancing photosynthetic rate of the Malaysian young ginger varieties Halia Bentong and Halia Bara under greenhouse conditions. These two varieties responded similarly to SA application.

### 2.2. Effect of SA Concentration on Plant Height, Leaf Area and Dry Weight

Foliar application of SA affected (*P* ≤ 0.05) total dry weight and plant leaf area. In both varieties, plant height was lower in treated plants than in controls, although the difference was not significant. Total dry weight and leaf area were greater in 10^–5^ M treated plants than in 10^−3^ M treated plants and the controls (Table 1). The highest total dry weight (8.68 g per plant) was observed in Halia Bentong, and the largest leaf area (719.2 cm^2^ per plant) was recorded in Halia Bara. These results are consistent with published reports. A recent study by Nagasubramaniam *et al.* [29] demonstrated that SA (7.2 × 10^−4^ M) increased plant height, leaf area, crop growth rate and total dry matter production in baby corn, while Jeyakumar *et al.* [30] reported that SA (10^–4^ M) enhanced dry matter production in black gram. Plant height and shoot and root dry weight were affected by SA application, but no significant differences were observed between treatments. Singh and Usha [31] also demonstrated that SA enhanced plant dry mass and Rubisco carboxylase activity in wheat.

**Table 1:** Effect of foliar SA application (10^−3^ and 10^−5^ M) on plant height, leaf area and dry weight of ginger parts.

Variety	SA(M)	Plant height	Leaf area	Leaf dry weight	Shoot dry weight	Root dry weight	Rhizome dry weight	Total dry weight
H.Bentong	Control	56.66 ± 6.75 ^a^	506.6 ± 15.6 ^c^	1.79 ± 0.53 ^b^	1.86 ± 0.67 ^a^	0.214 ± 0.05 ^a^	3.42 ± 0.66 ^a^	7.28 ± 1.51 ^ab^
	10^−5^	53.26 ± 5.51 ^a^	603.6 ± 16.14 ^abc^	2.31 ± 0.21 ^b^	2.1 ± 0.49 ^a^	0.24 ± 0.06 ^a^	4.03 ± 1.06 ^a^	8.68 ± 0.71 ^a^
	10^−3^	55.43 ± 4.55 ^a^	541.3 ± 62.02 ^c^	2.55 ± 0.65 ^b^	1.98 ± 0.21 ^a^	0.227 ± 0.035 ^a^	3.56 ± 0.78 ^a^	8.31 ± 1.09 ^a^
H.Bara	Control	49.24 ± 0.9 ^a^	592.4 ± 16.1 ^bc^	2.64 ± 0.29 ^b^	1.74 ± 0.49 ^a^	0.252 ± 0.068 ^a^	0.74 ± 0.11 ^b^	5.37 ± 0.68 ^b^
	10^−5^	48.5 ± 3.12 ^a^	719.2 ± 91.5 ^a^	3.72 ± 0.93 ^a^	2.21 ± 0.37 ^a^	0.281 ± 0.03 ^a^	0.99 ± 0.37 ^b^	7.2 ± 1.73 ^ab^
	10^−3^	48.36 ± 8.4 ^a^	673.9 ± 32.9 ^ab^	3.67 ± 0.70 ^a^	2.13 ± 0.23 ^a^	0.263 ± 0.06 ^a^	0.95 ± 0.25 ^b^	7.01 ± 1.17 ^ab^

Data are means of triplicate measurements ± standard deviation. Means not sharing a common single letter were significantly different at *P* ≤ 0.05. Plant height is expressed in cm, leaf area in cm^2^ per plant, and dry weight in g per plant.

SA is known to affect a range of plant functions. Previous studies have shown the involvement of SA in regulating growth processes such as stimulation of root formation in young shoots of ornamental plants [32] and increase of boll numbers in cotton after spraying at the flowering stage [33]. Conversely, Pancheva *et al.* [34] have reported that growth of barley seedling leaves and roots decreased when plants were treated with SA, with an even stronger effect observed when the concentration of SA was increased. Similarly, dose-dependent bud formation inhibition was reported in *Funaria hygrometrica* by Christianson and Duffy [35].

When applied in various quantities, phenolic compounds are well known to rapidly alter plant phenotypes and to influence plant growth from seed germination to senescence, either by enhancing or stimulating the natural growth regulatory system [1,36]. Our observations that stimulation of photosynthetic rate by SA was due to increases in activity within the leaf generally support previous findings and provide new information regarding mechanisms, levels of application and effective types of phenolic compounds. Our results suggest that increases in photosynthetic rate may have stimulated plant total dry weight production.

### 2.3. Nitrate Reductase Activity

**Figure 2:**
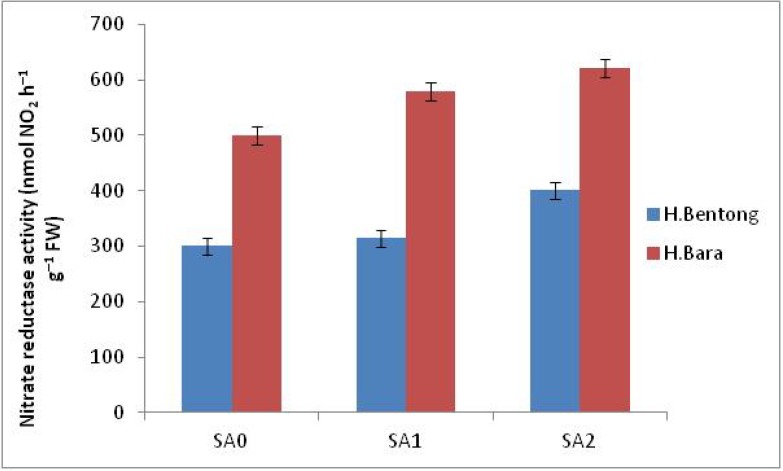
Nitrate reductase activity in Halia Bentong and Halia Bara treated with different concentrations of SA (SA0: control; SA1: 10^–3^ M; SA2: 10^–5^ M).

Spraying plants with SA, irrespective of concentration, generated significant increases in nitrate reductase activity in rhizomes (Figure 2). Application of 10^–5^ M SA was significantly superior to 10^–3^ M and no treatment, although lower nitrate reductase activity was observed in control plants. Nitrate reductase activity was higher in Halia Bara than in Halia Bentong for all treatments, including controls. In general, the highest nitrate reductase activity was recorded in Halia Bara sprayed with 10^–5^ M SA (621.9 nmol NO_2_ h^–1^ g^–1^ FW), while the lowest activity was observed in untreated Halia Bentong (300 nmol NO_2_ h^–1^ g^–1^ FW). This SA concentration-based effect on nitrite reductase activity may indicate that nitrite reductase activity was induced and/or enzyme degradation was prevented. Foliar application of Tween-20 generated a response that was statistically equal to that of the control. This finding was unexpected and suggests that an SA concentration of 10^–5^ M might induce nitrite reductase synthesis by mobilizing intracellular NO_3_^–^ and protect against nitrite reductase degradation *in vivo* in the absence of NO_3_^–^ [32]. 

Our results corroborate the findings of many other related studies. In particular, they are consistent with those of Fariduddin *et al.* [14], who found that low SA concentrations increased nitrite reductase activity in *Brassica juncea*, whereas higher concentrations were inhibitory. It can therefore be assumed that SA concentration plays an important role in regulating nitrate reductase activity, with lower concentrations enhancing nitrate reductase protein and higher concentrations decreasing it by affecting the above processes. Increases in nitrate concentration and in turn nitrate reductase activity due to exogenous SA treatment under normal growth conditions have been reported previously [7] and strongly support our observed results.

### 2.4. Chlorophyll Content

As shown in Table 2, SA significantly affected (*P* < 0.05) photosynthetic pigments (chlorophyll a, b and a+b). Chlorophyll concentrations significantly increased when SA concentration was increased from 10^–5^ M to 10^–3^ M. High concentrations of chlorophyll a, b and a + b were obtained in Halia Bara sprayed with 10^–3^ M SA: 279.93, 110.35 and 390.29 μg mL^−1^, respectively. A recent study found that a SA foliar spray increased chlorophyll a and b and carotenoid content in bean plants under normal field conditions [37]. Pancheva *et al.* [34] discovered that treatment of barley seedlings with SA for 7 d decreased chlorophyll content compared with untreated control plants. It is apparent from Table 1 that plants pretreated with 10^–5^ M SA exhibited higher chlorophyll contents. These results are in agreement with those obtained by other authors showing that SA significantly increases plant pigment content [38].

**Table 2:** Effect of foliar SA application (10^−3^ and 10^−5^ M) on chlorophyll content of ginger.

	H.Bentong			H.Bara		
	Control	SA 10^−5^ M	SA 10^−3^ M	Control	SA 10^−5^ M	SA 10^−3^ M
Chl a	181.95 + 20.85 ^d^	234.35 + 17.88 ^c^	256.23 + 2.60 ^b^	188.87 + 4.13 ^d^	251.79 + 3.43 ^b^	279.93 + 3.32 ^a^
Chl b	87.27 + 8.28 ^b^	102.46 + 25.88 ^a^	103.84 + 29.75 ^a ^	95.29 + 19.34 ^b^	96.17 + 12.91 ^b^	110.35 + 10.32 ^a^
Chl a+b	269.23 + 12.67 ^d^	336.81 + 12.02 ^c^	360.07 + 30.52 ^b^	284.17 + 16.73 ^d^	347.96 + 9.54 ^c^	390.29 + 7.08 ^a^

All analyses are the mean of triplicate measurements ±standard deviation; Results expressed in μg mL^−1^; Means not sharing a common single letter were significantly different at *P* ≤ 0.05.

### 2.5. Antioxidant Enzyme Activities and Proline Content

**Figure 3:**
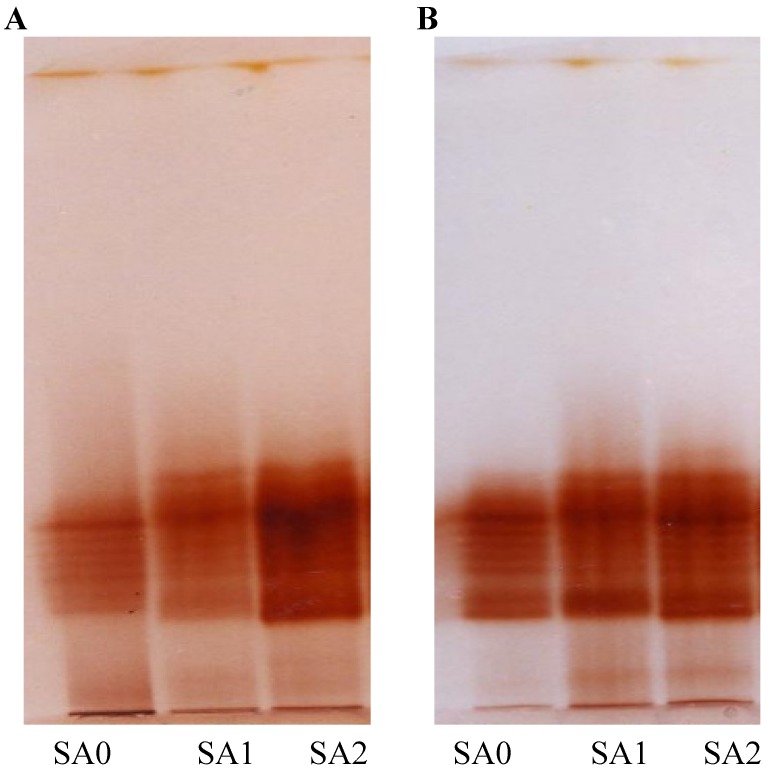
Gel image of peroxidase enzyme activity in Halia Bentong (**A**) and Halia Bara (**B**) treated with different concentrations of SA (SA0: control; SA1: 10^–3^ M; SA2: 10^–5^ M).

**Figure 4:**
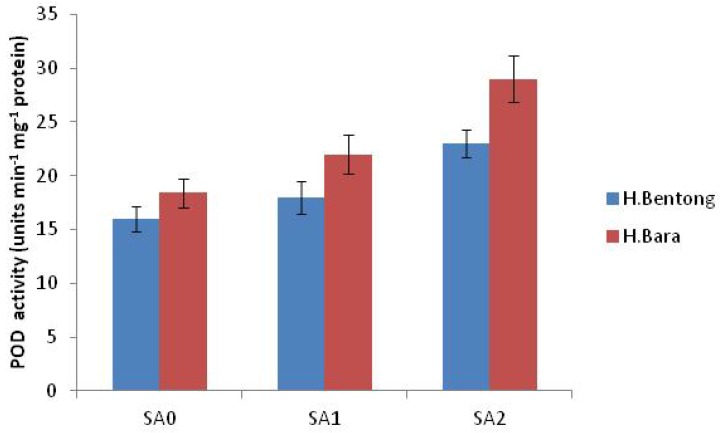
Peroxidase activity in Halia Bentong and Halia Bara treated with different concentrations of SA (SA0: control; SA1: 10^–3^ M; SA2: 10^–5^ M).

**Figure 5:**
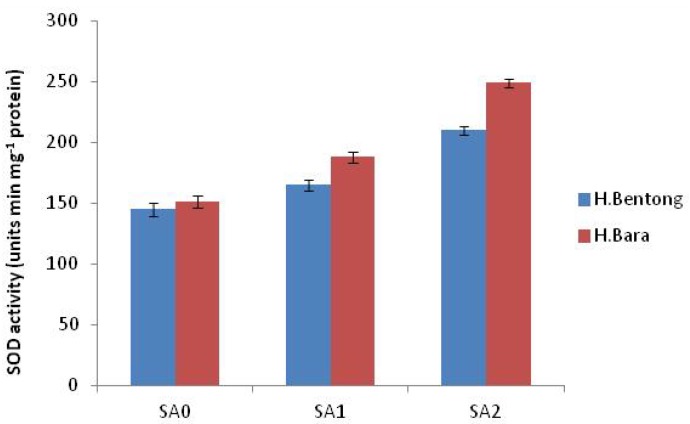
Superoxide dismutase (SOD) activity in Halia Bentong and Halia Bara treated with different concentrations of SA (SA0: control; SA1: 10^–3^ M; SA2: 10^–5^ M).

**Figure 6:**
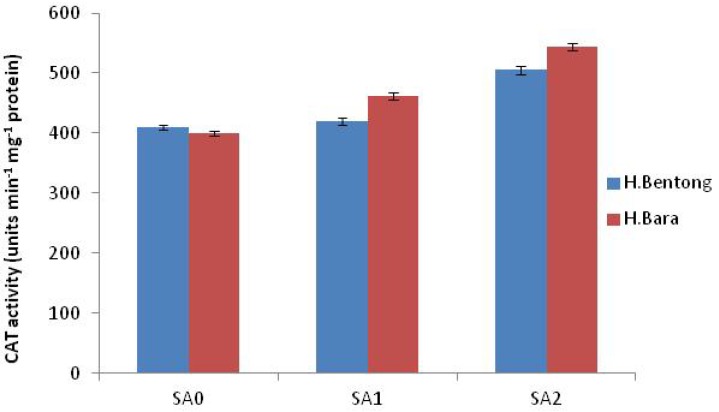
Catalase enzyme activity in Halia Bentong and Halia Bara treated with different concentrations of SA (SA0: control; SA1: 10^–3^ M; SA2: 10^–5^ M).

**Figure 7:**
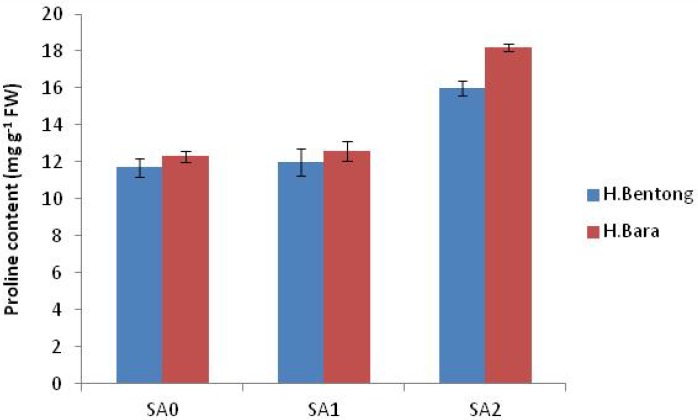
Proline content in Halia Bentong and Halia Bara treated with different concentrations of SA (SA0: control; SA1: 10^–3^ M; SA2: 10^–5^ M).

Exogenous application of SA increased antioxidant enzyme activities, with the maximum response generated in plants sprayed with 10^–5^ M SA. Significant increases were observed for POD (45.2%; Figure 3 and Figure 4), SOD (44.1%; Figure 5), CAT (20.1%; Figure 6) and proline (43.1%; Figure 7). Tween-20 application did not generate any significant response. Exogenous application of two different concentrations (10^–3^ and 10^–5^ M) of SA resulted in increased growth and physiological responses, with the best response generated from 10^–5^ M SA. Similar results have been obtained in *B. juncea* [14] and wheat [7].

**Figure 8:**
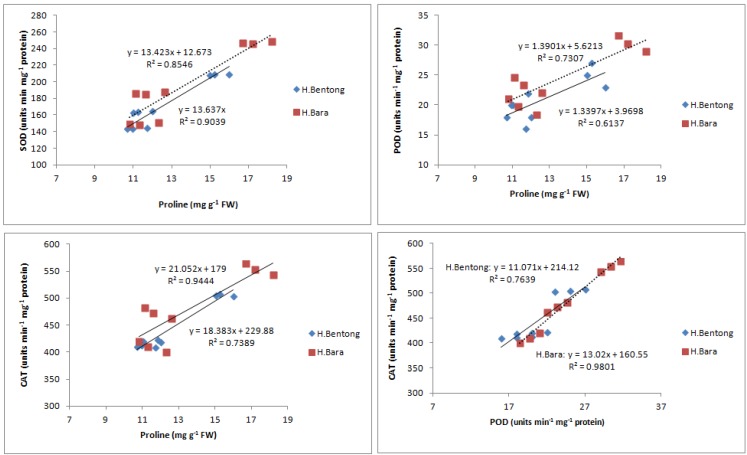
Correlations between proline content and superoxide dismutase (SOD), catalase (CAT) and peroxidase (POD) activities. * *P* < 0.05; ** *P* < 0.01.

After Rubisco, CAT is the most abundant soluble zinc-containing enzyme in C_3_ plant chloroplasts; it facilitates CO_2_ diffusion across chloroplast membranes by catalyzing the reversible hydration of dissolved CO_2_ entering the highly alkaline stromal environment [39] and maintains a constant supply of Rubisco. This enzyme’s concentration, and therefore its activity, is finely regulated at transcriptional and/or translational levels [40]. In our study, however, neither of these processes was favored by the application of 10^–5^ M SA, which increased CAT activity (Figure 6). Exogenous SA is known to increase stomatal conductance (Table 1); by maintaining a constant supply of CO_2_ for reduction by Rubisco [40], increased CAT activity in leaves is thus expected to increase photosynthetic efficiency and thereby net photosynthesis (Table 1). Exogenous SA increases pigment concentration and activates Rubisco and PEP carboxylase [31]. Enhancement of these characteristics ultimately increased net photosynthetic levels (Table 1), which increased plant growth, as reflected in the observed increases in dry mass per plant (Table 1). The higher SA concentration, however, may have caused permanent changes to cell membrane-level organization. These alterations would injure plant general metabolism and thereby reduce overall growth and photosynthetic attributes, a result observed in a study by Uzunova and Popova [41]. At the lower SA concentration, activities of antioxidant enzymes POD, SOD and CAT were elevated (Figure 4, Figure 5, Figure 6), in accordance with other studies [7]. This increased antioxidant enzyme activity might be due to SA’s regulatory role at transcriptional and/or translational levels. Proline enzyme activity was also influenced by SA treatment. Foliar application of 10^–3^ M SA significantly increased proline activity in both varieties, with the highest proline enzyme activity, 18.2 mg g^-1^ FW, recorded in Halia Bara treated with 10^–5^ M SA. As shown in Figure 7, proline activity was higher in Halia Bara than in Halia Bentong. Hayat *et al.* [7] reported that SA application increases plant proline content; this is in accordance with our results, where foliar SA application increased proline synthesis and thus its content in leaves. In addition to its action as an excellent osmolyte, proline stabilizes complex II electron transport [42], protein membrane and 3-D structure [43] and enzymes such as Rubisco and CAT [44], thereby increasing photosynthetic attributes. Foliar application of 10^-5^ M SA increased endogenous proline content, whereas the higher concentration may have reversed the phenomenon (Figure 7). Application of 10^–5^ M SA also resulted in higher net photosynthetic rates, which should lead to increased crop productivity (Table 1). As shown in Figure 8, proline, POD, SOD and CAT were significantly (*P* ≤ 0.05 and 0.01) and positively correlated in both varieties. Observed positive correlations among proline, SOD, CAT and POD activities suggest that increased SOD activity was accompanied by increases in CAT and POD activities because of the high demands of H_2_O_2_ quenching. Proline accumulation is reported to activate antioxidant defense mechanisms [45,46].

## 3. Experimental

### 3.1. Plant Material and Cultivation

Rhizomes of ginger varieties Halia Bentong and Halia Bara were sprouted for 2 weeks in 10-cm diameter pots filled with peat. Sprouted rhizomes were transferred to polyethylene bags filled with a soilless mixture medium containing burnt rice husks and coco peat (1:1). The plants were grown in a glasshouse at the University Putra Malaysia (UPM) glasshouse complex, with seedlings raised in specially constructed growth houses under a 12-h light photoperiod with an average photosynthetic photon flux density of 310 μmol m^–2^ s^–1^. Day and night temperatures were 30 ± 1.0 °C and 20 ± 1.5 °C, respectively, and relative humidity was about 70–80%. When the ginger seedlings were at the second leaf stage, they were sprayed with two concentrations (10^–3^ and 10^–5^ M) of a salicylic acid (SA) solution containing 2-hydroxybenzoic acid, 100 μL dimethyl sulfoxide, and 0.02% polyoxyethylene sorbitan monolaurate (Tween-20; Sigma-Aldrich, Selangor, Serdang, Malaysia) at pH 6.5. Control plants were sprayed with a similar solution lacking SA. Leaves were sprayed once weekly, early in the morning, for one month. 

### 3.2. Photosynthetic Rate, Stomatal Conductance and Transpiration Measurements

After spraying, photosynthetic rate, stomatal conductance and transpiration rate of fully expanded leaves were measured for 6 d using a LICOR-6400 portable photosynthesis system (LI-COR, Lincoln, NE, USA).

### 3.3. Plant Biomass and Leaf Area Measurements

Plants were harvested 4 weeks after planting in polyethylene bags. Plant parts were separated, dried at 70 °C for 72 h, and weighed on an electronic weighing scale (Mettler-Toledo Model B303-S, Geneva, Switzerland). Leaf area per plant was measured using the leaf area meter Model LI–3100 (LICOR Inc., Lincoln, NE, USA).

### 3.4. Chlorophyll Measurement

To measure chlorophyll concentration, fresh leaves (200 mg) were ground using a mortar and pestle and immersed in 100% acetone (10 mL). Samples were wrapped in aluminum foil and homogenized with a B-Braun-type homogenizer at 1,000 rpm for 1 min. The homogenate was filtered through two layers of cheesecloth, and then centrifuged at 2500 rpm for 10 min. After separation, the absorbance of the supernatant was measured in quartz cuvettes against a blank of 100% acetone at two wavelengths—662 nm and 645 nm, the peak absorbances of chlorophyll a and chlorophyll b, respectively. Total amounts of chlorophyll a and chlorophyll b were then calculated according to the formulas of Lichtenthaler and Wellburn [47].

### 3.5. Nitrate Reductase Activity

Leaves from different treatments were collected and cut into small pieces. Leaf pieces (about 500 mg) were incubated separately in a medium containing 1 M potassium nitrate (1 mL), 0.2 M phosphate buffer (pH 7.5, 2 mL) and 0.5% Triton X-100 (2 mL) for 1 h. One milliliter of reaction mixture was transferred to another test tube and mixed with 1% sulfanilamide in 2 N hydrochloric acid (1 mL) and 0.2% NEEDA (*N*-1-napthylethylenediamine dihydrochloride, 1 mL). Using a mixture of NEEDA (1 mL) as a blank, absorbance was recorded at 540 nm in a LKB Biochem Ultra Spectrophotometer [48]. A standard curve was prepared using different concentrations of potassium nitrite. Enzyme activity was expressed as nmol of NO_2_ liberated per hour per gram FW.

### 3.6. Proline Determination

Fresh leaf samples (1 g) were ground in a mortar after addition of a 3% (w/v) aqueous sulfosalicylic acid solution (10 mL). The homogenate was filtered through two layers of glass-fiber filter, and the clear filtrate was then used in the assay. Ninhydrin reagent and glacial acetic acid (1 mL each) were added to 1 mL of the filtrate. Test tubes containing the reaction mixture were covered and incubated in a boiling water bath for 1 h, and then placed in a room temperature (21°C) water bath for 5 min to terminate the reaction. Absorbance readings were taken immediately at 546 nm [49]. Proline concentration was determined from a standard curve and calculated on a fresh weight basis (mg g^–1^ FW).

### 3.7. Antioxidant Enzyme Assays

The nitroblue tetrazolium (NBT) method was used for determination of SOD activity [18]. One unit of SOD activity was defined as the amount of enzyme required to produce 50% inhibition of NBT reduction at 560 nm. The methods of Fu and Huang [18] were used to determine POD and CAT activities. For POD, guaiacol oxidation was measured by monitoring increase in absorbance at 470 nm for 1 min. For CAT, H_2_O_2_ decomposition was measured by recording decline in absorbance at 240 nm for 1 min. One unit of CAT or POD activity corresponded to an absorbance change of 0.01 units per min. The activity of each enzyme was expressed on a protein basis. Three replicates per treatment and variety were performed at midday using the youngest fully expanded leaves of different individuals.

### 3.8. Statistical Analysis

Data were analyzed using Statistical Analysis System (SAS) version 9.0 (2002). Treatment means were separated using Duncan’s multiple range test. Experimental results were expressed as means of three replicates ± standard deviation. A *P*-value ≤ 0.05 was regarded as significant.

## 4. Conclusions

This investigation has highlighted the usefulness of SA in plant growth regulation. One of the more significant findings to emerge from this study is that foliar application of SA can improve plant physiological efficiency, including photosynthetic rate, and can enhance effective partitioning of accumulates from source to sink. Treatment with SA resulted in higher photosynthetic rates and increased plant biomass. Most obviously, antioxidant enzyme activities and proline content were enhanced in ginger plants treated with the lower SA concentration (10^–5^ M). In addition, exogenous application of two different concentrations (10^–3^ and 10^–5^ M) of SA resulted in increased growth and physiological responses, with the best response generated using 10^–5^ M SA. Our results support the idea that low SA concentrations (10^–5^ M) induce nitrite reductase synthesis by mobilizing intracellular NO^3–^ and provide protection to nitrite reductase degradation *in vivo* in the absence of NO^3–^. Positive correlations observed among proline, SOD, CAT and POD activities in the studied varieties suggest that increased SOD activity was accompanied by increases in CAT and POD activities because of the high demands of H_2_O_2_ quenching. By boosting photosynthetic rate in response to enhanced antioxidant enzyme activities, it therefore appears that SA can generally be used as a growth regulator to enhance plant growth and yield.
